# Antibiotic resistance and multidrug‐resistant efflux pumps expression in lactic acid bacteria isolated from pozol, a nonalcoholic Mayan maize fermented beverage

**DOI:** 10.1002/fsn3.304

**Published:** 2015-11-09

**Authors:** Maria del Carmen Wacher‐Rodarte, Tanya Paulina Trejo‐Muñúzuri, Jesús Fernando Montiel‐Aguirre, Maria Elisa Drago‐Serrano, Raúl L. Gutiérrez‐Lucas, Jorge Ismael Castañeda‐Sánchez, Teresita Sainz‐Espuñes

**Affiliations:** ^1^ Depto de Alimentos y BiotecnologíaFacultad de QuímicaUNAMCiudad Universitaria Coyoacán04510MéxicoDistrito FederalMéxico; ^2^ Depto de BioquímicaFacultad de QuímicaUNAMCiudad Universitaria Coyoacán04510MéxicoDistrito FederalMéxico; ^3^ Depto. Sistemas BiológicosUAM‐XochimilcoCalzada del Hueso No.1100, Coyoacan04960MexicoDistrito FederalMéxico

**Keywords:** Antibiotic resistance, efflux pumps, ethidium‐bromide, lactic‐acid‐bacteria, Mayan‐pozol

## Abstract

Pozol is a handcrafted nonalcoholic Mayan beverage produced by the spontaneous fermentation of maize dough by lactic acid bacteria. Lactic acid bacteria (LAB) are carriers of chromosomal encoded multidrug‐resistant efflux pumps genes that can be transferred to pathogens and/or confer resistance to compounds released during the fermentation process causing food spoiling. The aim of this study was to evaluate the antibiotic sensibility and the transcriptional expression of ABC‐type efflux pumps in LAB isolated from pozol that contributes to multidrug resistance. Analysis of LAB and *Staphylococcus* (*S*.) *aureus* ATCC 29213 and ATCC 6538 control strains to antibiotic susceptibility, minimal inhibitory concentration (MIC), and minimal bactericidal concentration (MBC) to ethidium bromide were based in “standard methods” whereas the ethidium bromide efflux assay was done by fluorometric assay. Transcriptional expression of efflux pumps was analyzed by RT‐PCR. LAB showed antibiotic multiresistance profiles, moreover, *Lactococcus* (*L*.) *lactis* and *Lactobacillus* (*L*.) *plantarum* displayed higher ethidium bromide efflux phenotype than *S*. *aureus* control strains. Ethidium bromide resistance and ethidium bromide efflux phenotypes were unrelated with the overexpression of *lmrD* in *L*. *lactics*, or the underexpression of *lmrA* in *L*. *plantarum* and *norA* in *S*. *aureus*. These findings suggest that, moreover, the analyzed efflux pumps genes, other unknown redundant mechanisms may underlie the antibiotic resistance and the ethidium bromide efflux phenotype in *L*. *lactis* and *L*. *plantarum*. Phenotypic and molecular drug multiresistance assessment in LAB may improve a better selection of the fermentation starter cultures used in pozol, and to control the antibiotic resistance widespread and food spoiling for health safety.

## Introduction

Pozol is a handcraft traditional nonalcoholic beverage produced from fermented maize dough and consumed mainly in southeastern Mexico, in the Maya region, as an important component of their diet (Ulloa et al. 1996). Pozol production entails a complex fermentation process of more than 40 different species of lactic acid bacteria, yeasts, and fungi (Wacher et al. 1993).

Analysis of *Escherichia* (*E*.) *coli* pathotype strains and other intestinal bacteria isolated from pozol indicated that a 60% were resistant to one antibiotic whereas 4% were antibiotic multiresistant (Sainz et al. 2001). In lactic acid bacteria (LAB), antibiotic resistance has been ascribed to multidrug resistant (MDR) efflux pumps involved in the expulsion of structurally unrelated compounds antibiotics, biocides, toxic agents like ethidium bromide (Mazurkiewicz et al. 2005). Efflux pumps including the chromosomally encoded ABC‐type transporters LmrA and LmrCD described in *Lactococcus* (*L*.) *lactis* (Poelarends et al. 2002; Lubelski et al. 2006). LmrA transporter is associated with the resistance to wide variety of clinically relevant antibiotics (Poelarends et al. 2002), whereas LmrCD confers resistance to toxic compounds like daunomycin, cholate, and ethidium bromide (Lubelski et al. 2006). In *Lactobacillus* (*L*.) *brevis* and *L*. *plantarum,* the ABC transporter HorA confer resistance to toxic compounds generated during beer fermentation (Ulmer et al. 2000, 2002; Sakamoto et al. 2001). Lactic acid bacteria especially *Lactobacillus spp*. with resistance to toxic compounds are regarded as spoil strains for the production of fermented beverages like beer (Sakamoto and Konings 2003). Moreover, LAB are carriers of antibiotic‐resistant genes that can be transferred to other bacteria including human pathogens (Toomey et al. 2009, 2010). Thus, analysis of MDR genes in LAB is relevant not just for preventing the spoiling of foods and beverages, but also to control the potential dissemination of resistance‐associated genes to harmless bacteria from the intestinal microbiota and even foodborne pathogens. ABC efflux pumps are extensively described in *Lactococcus lactis*, so this may be a first approach to study this system in pozol LAB, however, other efflux transporters (Piddock 2006) will be investigated in future work.

The aim of this study was to evaluate the antibiotic resistance and the expression of the ABC‐type pump genes that contributes to the multidrug resistance in LAB isolated from pozol in order to achieve a better selection of the fermentation starter cultures.

## Material and Methods

### Selection of strains

Strains belonging to *Weissella, Lactococcus, Lactobacillus, Leuconostoc, Streptococcus,* and *Enterococcus* genus were obtained from Dr. Wacher's pozol strains collection. *Staphylococcus aureus* ATCC 29213 was used as control strain.

### Microbiologic procedures

Reagents and media were obtained from Sigma Chemical Co., St. Louis, Mo., and BD Bioscience, Sparks, MD. APT broth was used for *Weisella, Lactococcus, Lactobacillus,* and *Leuconostoc* cultures, and was incubated at 30°C for 24 h. MRS broth was used for *Enterococcus* and *Streptococcus* cultures, and incubated at 37°C for 24 h.

### Susceptibility testing

Antimicrobial Disk Susceptibility Tests in accordance with the procedures outlined by Clinical and Laboratory Standards Institute (CLSI 2006) were performed for the pozol strains and controls. APT agar and MRS agar were used for this method. Inocula were adjusted to 1.5 × 10^8^ CFU/mL (0.5 McFarland). BD BBLTM Sensi‐Discs (Becton Dickinson and Company, Sparks, MD) were used. The antibiotics tested were: Ampicilin (AM), Penicilin (PE), Dicloxacilin (DC), Cloxacilin (CX), Cefotaxime (CFX), Cefalotine (CF), Ciprofloxacin (CPF), Clindamicin (CLM), Eritromicin (E), Tetraciclin (TE), Gentamicin (GE), Netilmicin (NET), Kanamicin (K), Neomicin (N), Trimetoprim/Sulfametoxazol (SXT), Vancomicin (VA), and Chloramphenicol (C). Plates were incubated as mention before. Determinations were performed by triplicate. The definition for R/S character for antibiotic susceptibility followed CLSI criteria (CLSI 2006).

### Minimal inhibitory or bactericide concentration to Ethidium bromide

#### Ethidium bromide (EB) efflux assay

The ability of some microorganisms to efflux ethidium bromide, which inside the cell intercalates with double‐stranded nucleic acids, thus determining fluorescence increase when properly excited. Ethidium bromide is a substrate for a variety of efflux pumps so, once inside the cell, it is extruded with the result of decreasing the overall measurable fluorescence.

For EB MICs, 50 mL of suitable broth were inoculated with a 24 h overnight culture adjusted to 0.5 McFarland, and variable concentrations of ethidium bromide (5, 10, 20, 40, and 80 μg/mL) were added to each test tube. Culture media and EB tubes were used as negative controls and inoculated tubes with each strain without EB were used as positive controls. Tubes were incubated at 30°C or 37°C as described and growth (turbidity) was measured during 24 h, 48 h, and 1 week later. MIC was defined as the lowest concentration of EB in which no growth was present after 48 h of incubation time.

#### Ethidium bromide uptake

For this test, a modification of the procedure described by Kaatz et al. (2000) and Patel et al. (2010) was used. Cells were grown overnight in suitable broth and adjusted to 0.4 McFarland. Ten μg/mL of EB (final concentration) was added and incubated for 25 min at room temperature. Cells were harvested by centrifugation (7000 ×g) and washed with fresh culture media, and then 4 mL of suitable broth was added. The suspension was maintained at 30°C or 37°C, and the fluorescence of aliquots was determined at frequent intervals of time (5–30 min) in a Hitachi F‐4500 fluorometer, Tokyo (excitation wavelength, 540 nm; emission wavelength 545 nm).

Expression of efflux with significant differences was in accordance with that described by Patel et al. (2010) where at least a 20% difference of the fluorescence intensity has to be achieved between the tested strains and the control.

#### Gene expression

Total RNA was isolated using the SV Total RNA Isolation System (Promega, Madison, WI) following the manufactures instructions with additional modifications, lysozyme (10 mg/mL) and lysostaphin (0.5 mg/mL) were added to a final volume of 100 μL in TE buffer to assure all the bacterial walls were disrupted. RNA concentration was determined using a 2000 Nanodrop spectrophotometer (Thermo Scientific, Wilmington, DE) and was stored at −70°C until used for RT‐PCR assay.

#### RT‐PCR analysis

*Lactococcus lactis secY* and *lmrD*,* Lactobacillus plantarum secY* and *lmrA, Staphylococcus aureus secY* and *norA* mRNAs were examined by one‐step reverse transcription (Qiagen OneStep RT‐PCR Kit, Valencia, CA.) following the manufacturer's instructions.

A total of 0.5 μg was reverse transcribed with Superscript II reverse transcriptase at 50°C for 30 min; followed by amplification with specific primers listed in Table 1. An initial step of 15 min at 95°C followed by 30 amplification cycles consisting of 30 sec of denaturation at 94°C, 30 sec of annealing temperature (Table 2), and 1 min of extension at 72°C were used. After amplification, RT‐PCR products were analyzed on 1.5% agarose gels, and bands were visualized after ethidium bromide staining.

**Table 1:** Primers used in this study

Gen	Oligonucleotide sequences	Amplicon (bp)	Ref
	*Staphylococcus aureus*		
*secY*	F: ATCCCCAAGGTTCTCAAGGT	174	This study
	R: CACCTTGTTTTGCCCATTCT		
*norA*	F: TTATATCGCCGTTTGGTGGT	246	
	R: TCGCTGACATGTAGCCAAAG		
	*Lactococcus lactis*		
*secY*	F: GTGGTCAAAACAAGGGGAAA	217	This study
	R: TTGTTCACCCATCCAAGTGA		
*lmrD*	F: GGCAACTTCACATGCTGCTA	232	
	R: AGAGGTGAAACGAGCAAGGA		
	*Lactobacillus plantarum*		
*secY*	F: GCCGGGGTTATTCCTGTTAT	180	This study
	R: GAACGTGAAGAGCACGATCA		
*lmrA*	F: CTAACGCTTTTCCGCAAGTC	184	
	R: GCTAAAGCATCTTGGCGTTC		

**Table 2:** Annealing temperatures

Microorganism	Gen	°C
*Staphylococcus aureus*	*secY*	63.5
	*norA*	63.5
*Lactococcus lactis*	*secY*	55.0
	*lmrD*	63.5
*Lactobacillus plantarum*	*secY*	60.0
	*lmrA*	52.0

#### Densitometry analysis

Digital gel photographs of the stained RT‐PCR products were taken under UV exposure by using a Kodak EDAS (Eastman Kodak Company, Molecular Imaging Systems, NY, USA) 290 System. Amplicons (cDNA bands) were determined as the integrated area (pixels) of the band intensities by densitometric analysis with Kodak Digital Science1D 3.6 software (Eastman Kodak Company, Molecular Imaging Systems, NY, USA). The numerical values for cDNA band intensities were corrected with the values for the sec Y bands, as the *secY* gene is expressed at a relatively constant level in cells and is commonly used in semiquantitative RT‐PCR systems to assess the relative efficiency of each individual PCR. Tenfold logarithmic dilutions of the cDNA mixture were used to verify the linear correlation between the intensity (pixels) of the bands and the initial amount of cDNA.

## Results

Antibiogram tests were done as first step to evidence the sensibility or resistance of acid lactic bacteria to conventional antibiotics. According to CLSI susceptible antimicrobial susceptibility test, interpretive category (S) implies that isolates are inhibited by the usually achievable concentrations of the antimicrobial agent when the recommended dosage is used. The intermediate category (I) includes isolates for which response rates may be lower than susceptible isolates. The resistant category (R) implies that isolates are not inhibited by the usually achievable concentrations of the antimicrobial agent present in the commercial disks used. As shown in Table 3, *S*. *aureus* control strains had sensibility to penicillins, cephalosporins, erythromycin, tetracycline, kanamycin (ATCC 25923 and ATCC 6538), vancomycin, and chloramphenicol. Moreover, S*. aureus* showed resistance (R) to ciprofloxacin (*S*. *aureus* 29213); clindamycin, gentamicin, and neomycin or intermediate resistance (IR) to ciprofloxacin (*S*. *aureus* 25923 and ATCC 6538), netilmicin, kanamycin (*S*. *aureus* ATCC 29213), and sulfamethoxazole‐trimethoprim.

**Table 3:** Antibiogram tests of *Staphylococcus aureus* control strains and lactic acid bacteria strains

Strain	1				2		3	4	5	6	7				8	9	10
	AM	PE	DC	CX	CFX	CF	CPF	CLM	E	TE	GE	NET	K	N	SXT	VA	C
*S. aureus* ATCC25923	S	S	S	S	S	S	I	R	S	S	R	I	S	R	I	S	S
*S. aureus* ATCC6538	S	S	S	S	S	S	I	R	S	S	R	I	S	R	I	S	S
*S. aureus* ATCC29213	S	S	S	S	S	S	R	R	S	S	R	I	I	R	I	S	S
*E. italicus* A	S	S	S	S	S	S	S	S	S	S	R	S	S	R	R	S	S
*E. italicus* B	S	S	S	S	S	S	S	S	S	S	R	S	S	R	R	S	S
*S. bovis*	R	R	S	I	S	R	R	I	S	S	R	R	R	R	R	S	S
*W. confusa* A	R	S	R	R	R	R	R	I	S	S	R	S	R	R	R	R	S
*W. confusa* B	S	R	R	R	S	R	R	R	S	I	R	S	R	S	I	R	S
*L. plantarum* A	S	S	R	R	S	R	R	R	S	S	R	S	R	R	I	R	S
*L. plantarum* B	S	S	R	R	I	R	R	R	S	I	R	S	R	S	R	R	S
*L. lactis*	S	S	R	R	S	R	R	I	S	S	I	S	S	R	R	I	S
*L. mesenteroides* A	S	S	R	R	S	S	R	S	S	S	I	S	R	S	R	R	S
*L. mesenteroides* B	S	S	R	R	S	I	R	I	S	I	R	I	R	R	R	R	S

(S) Sensible, (R) Resistant, (I) Intermediate resistance.

*Enterococcus italicus* strains were sensible to all antibiotics except gentamicin, neomycin, and sulfamethoxazole‐trimethoprim.

*Streptococcus bovis* showed (1) sensibility to dicloxacillin, cefotaxime, erythromycin, tetracycline, vancomycin, and chloramphenicol; (2) resistance to ampicillin, penicillin, cefalotin, ciprofloxacin, all aminoglycosides tested and sulfamethoxazole‐trimethoprim; and (3) intermediated resistance to cloxacillin and clindamycin.

*Weisella confusa* showed (1) sensibility to ampicillin (strain B), penicillin (strain A); cefotaxime (strain B), erythromycin, tetracycline (strain A), netilmicin, neomycin (strain B), chloramphenicol; (2) resistance to ampicillin (strain A), penicillin (strain B), dicloxacillin, cloxacillin, cefotaxime (strain A), cefalotin, ciprofloxacin, clindamycin (strain B), gentamicin, kanamycin, neomycin (strain A), sulfamethoxazole‐trimethoprim (strain A), vancomycin; and (3) intermediate resistance to clindamycin (strain A), tetracycline (strain B), Sulfamethoxazole‐trimethoprim (strain B) (Table 3).

*Lactobacillus plantarum* showed (1) sensibility to ampicillin, penicillin, cefotaxime (strain A), erythromycin, tetracycline (strain A), netilmicin, neomycin (strain B), chloramphenicol; (2) resistance to dicloxacillin, cloxacillin, cefalotin, ciprofloxacin, clindamycin, gentamicin, kanamycin, neomycin (strain A), sulfamethoxazole‐trimethoprim (strain B), vancomycin; and (3) intermediate resistance to cefotaxime (strain B), tetracycline (strain B) and sulfamethoxazole‐trimethoprim (strain A).

*Lactococcus lactis* had (1) sensibility to ampicillin, penicillin, cefotaxime, erythromycin, tetracycline, netilmicin, kanamycin, chloramphenicol; (2) resistance to dicloxacillin, cloxacillin, cefalotin, ciprofloxacin, neomycin, sulfamethoxazole‐trimethoprim; and (3) intermediate resistance to clindamycin, gentamicin, and vancomycin.

*Leuconostoc mesenteroides* showed (1) sensibility to ampicillin, penicillin, cefotaxime, cefalotin (strain A), clindamycin (strain A), erythromycin, tetracycline (strain A), netilmicin (strain A), neomycin (strain A), chloramphenicol; (2) resistance to dicloxacillin, cloxacillin, ciprofloxacin, gentamicin (strain B), kanamycin, neomycin (strain B), sulfamethoxazole‐trimethoprim, vancomycin; and (3) intermediate resistance to cefalotin (strain B), clindamycin (strain B), tetracycline (strain B), gentamicin (strain A) and netilmicin (strain B).

### Sensibility test to ethidium bromide

Once the antimicrobial resistance to conventional antibiotics is estimated, the test for efflux phenotype was evidenced by assessing the sensibility or resistance to ethidium bromide, a DNA intercalating agent. Minimal inhibitory concentration (MIC) and minimal bactericidal concentration (MBC) to ethidium bromide from strains are summarized in the Table 4. As shown, *E*. *italicus* and *S*. *bovis* strains, showed MIC and MBC values twice or even lower in comparison with those from *S*. *aureus* control strains. Instead, *W*. *confusa*,* L*. *plantarum*,* L*. *lactis,* and *L*. *mesenteroides* showed MIC and MBC values twice or even greater than those from the *S*. *aureus* control strains.

**Table 4:** Assays of MIC and MBC to ethidium bromide

Strain	MIC (μg/mL)	MBC (μg/mL)
*S. aureus* ATCC25923	5	40
*S. aureus* ATCC6538	10	20
*S. aureus* ATCC29213	5	20
*E. italicus* A	<5	<5
*E. italicus* B	<5	10
*S. bovis*	<5	10
*W. confusa* A	40	>80
*W. confusa* B	40	>80
*L. plantarum* A	>80	>80
*L. plantarum* B	>80	>80
*L. lactis*	40	>80
*L. mesenteroides* A	>80	>80
*L. mesenteroides* B	>80	>80

MIC, minimal inhibitory concentration; MBC, minimal bactericidal concentration.

**Figure 1:**
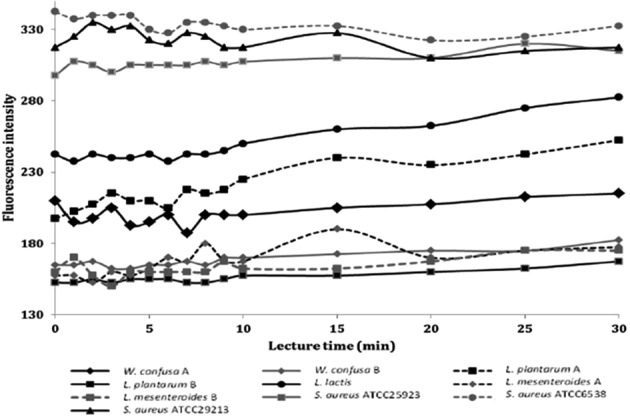
Accumulative test of ethidium bromide in bacterial strains with significant differences in fluorescence intensity in regard with the *Staphylococcus aureus* control strains assessed for five minutes each during thirty minutes.

Accumulative test of ethidium bromide was done given that this DNA intercalating toxic agent is used as substrate to detect phenotypically the expression of efflux pumps (Patel et al. 2010). In comparison with *S*. *aureus* control strains, accumulation of ethidium bromide was lower in *L*. *lactis*,* L*. *plantarum* (strain A), and *W*. *confusa* (strain A) or even lower in *W*. *confusa* (strain B), *L*. *plantarum* (strain B), and *L*. *mesenteroides* (strain B) (Fig. 1).

Agarose gel analysis of mRNA encoding efflux pumps amplified by RT‐PCR.

**Figure 2:**
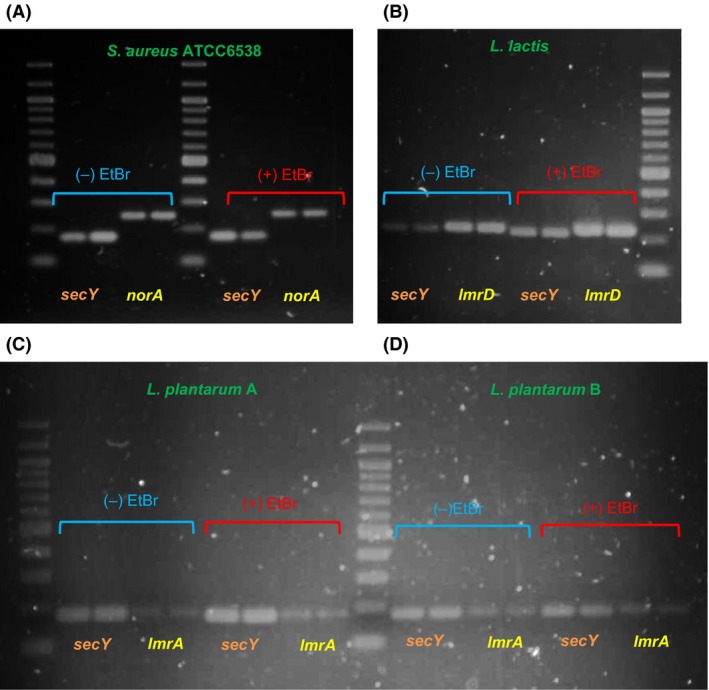
Agarose gel analysis of RT‐PCR products: (A) *Staphylococcus aureus* ATCC6538 control strain; (B) *Lactococcus lactis*;*Lactobacillus plantarum* strain A (C) and strain B (D).

Accumulation of ethidium bromide led us to the molecular characterization of efflux pumps in acid lactic bacteria. Efflux pumps have been more studied in *L*. *lactis* and *L*. *plantarum* strains, therefore they were selected to analyze the mRNA amplification by RT‐PCR encoding efflux pumps associated with antimicrobial multiresistance**.** Each strain showed two bands with divergent intensity indicating differential mRNA expression without or with ethidium bromide (Fig. 2).

**Figure 3:**
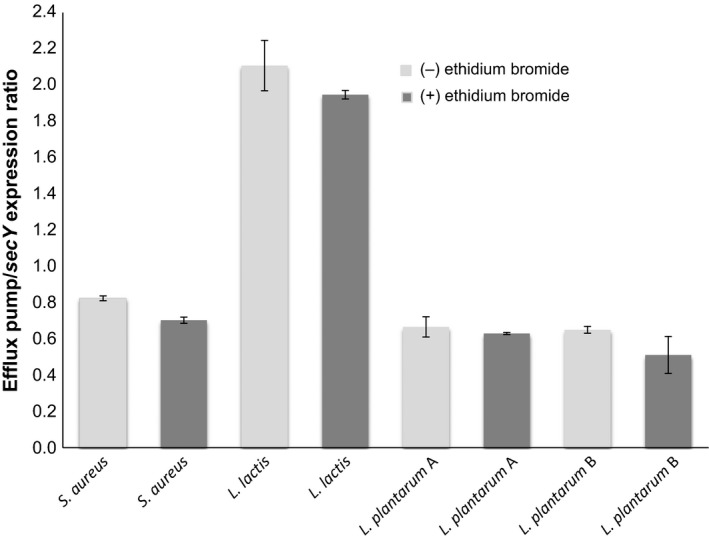
mRNA expression ratios of efflux pumps from each bacterial strain relative to *sec Y* mRNA expression with *Staphylococcus aureus* ATCC 6538 as control strain. Data are depicted as mean values plus standard deviation (SD).

Quantitative analysis by densitometry was done to estimate significant differences on the relative expression from the particular mRNA product of acid lactic bacteria regarding the constitutive expression of *secY* mRNA included as control. As shown in the Figure 3, no differences on the relative mRNA expression without or with ethidium were found in all strains. Thus, ethidium bromide is not a determining factor for their expression. In comparison with the relative *norA* mRNA expression of *S*. *aureus* ATCC 6538 control strain, no differences were found on the relative *lmrA* mRNA expression of *L*. *plantarum*. Moreover, relative mRNA expression found in *S*. *aureus* and *L*. *plantarum* was less than 1.0 in regard with the constitutive expression of *secY* control. Instead, significant differences were found concerning the relative *lmrD* mRNA expression of *L*. *lactis* in comparison with that observed in *S*. *aureus* and *L*. *plantarum* strains without and with ethidium bromide.

## Discussion

According to the results, most acid lactic bacteria strains (except *E*. *italicus*) displayed a profile of multiresistance as they showed (1) resistance (or intermediate resistance) to a greater antibiotic number; and (2) the antibiotic resistance was distributed in more than three antibiotic families by comparison with the *S*. *aureus* control strains.

Moreover, to exhibit resistance to antibiotics, *Lactococcus*,* Lactobacillus*,* Leuconostoc,* and *Weissella genus*, displayed higher resistance to ethidium bromide as well lower ethidium bromide accumulation in comparison with *S. aureus* control strains. In *L*. *lactis*, the resistance to antibiotics and to ethidium bromide as well as the high ethidium bromide efflux were independent from the overexpression of *lmrD* mRNA (Figs. 2, 3). In *L*. *lactis*,* lmrD* gene encodes D protein subunit of the heterodimeric ABC transporter LmrCD responsible for the efflux of a wide spectrum of toxic agents **(**ethidium bromide, daunomycin, cholate, acid bile**),** but not to some antibiotics **(**tetracycline kanamicin, chloramphenicol) (Lubelski et al. 2006; Zaidi et al. 2008). Transcriptional *lmrC and lmrD* gene expression is under the control of *lmrR* that encodes a LmrR repressor protein that in the absence of toxic compounds it binds to the promoter regions of the *lmrCD* genes to repress their transcription (Agustiandari et al. 2008). In the presence of toxic compounds, LmrR is released from the promoter regions to induce the transcription of the *lmrCD* genes (Agustiandari et al. 2008). In this study, molecular mechanism underlie the constitutive *lmrD* mRNA overexpression without or with ethidium bromide may have resulted from different modes of binding of LmrR to *lmrR* and *lmrCD* control regions resulting in the generation of different transcripts that encode different structural genes either with or without the *lmrR* transcriptional regulator gene (Agustiandari et al. 2011; Takeuchi et al. 2014). Another presumable mechanism may include defective LmrR that is unable to bind the promoter/operator region of the lmrCD to accomplish their repression (Lubelski et al. 2006).

In this study, the mRNA expression of *lmrA* was analyzed, an ATP‐dependent multidrug resistance transporter of the ABC family conferring resistance on beer spoilage *Lactobacillus* strains to toxic hop compounds (Ulmer et al. 2000; Sakamoto et al. 2001). In *L*. *plantarum* strains, resistance to antibiotics and to ethidium bromide as well the high ethidium bromide efflux phenotype were accompanied by a lower expression of *lmrA* mRNA. Moreover, *lmrA* mRNA expression was independent from the presence of ethidium bromide in spite of this is a substrate for LmrA protein transporter as described in *L*. *plantarum* for a highly homolog gene *horA* (Ulmer et al. 2000, 2002; Sakamoto et al. 2001). These findings indicate that in the analyzed *L*. *plantarum* strains, LmrA‐independent ethidium bromide efflux phenotype may have resulted by alternative mechanisms including an altered fluidity and composition of the cytoplasmic membrane as well‐modified cell wall composition of lipoteichoic acids as described in *L*. *brevis* (Behr et al. 2006).

In comparison with some lactic acid strains, *S*. *aureus* control strains displayed higher sensibility to ethidium bromide and weaker ethidium bromide efflux phenotype. Analysis of sensibility to ethidium bromide in *S*. *aureus* ATCC 25293 has been also described previously (Couto et al. 2008). In this assay, phenotypical features of resistance to ethidium bromide found in *S*. *aureus* strains were unrelated with the *norA* mRNA expression, which was unaltered without or with ethidium bromide. As it is known, *norA* provides resistance to fluoroquinolones and even, *norA* overexpression is associated with multidrug resistance of mutants obtained by the exposition of parental strains with increasing concentrations of biocides and toxic dyes (Bhateja et al. 2006; Huet et al. 2008). The findings of this assay may reflect the wide array of *norA*‐independent mechanisms that in the case of *S*. *aureus* have been described more than 10 efflux pumps systems (Andersen et al. 2015). Importantly, although these pumps show different substrate specificity, most of them are capable of extruding compounds of different chemical classes, thus providing the cell with the means to develop a MDR phenotype or to survive in a hostile environment (Poole 2007).

In conclusion, the findings showed that the antibiotic resistance and the efflux phenotype were independent from the overexpression of *lmrD* mRNA in *L*. *lactis* or the lower expression of *lmrA* or *norA* in *L*. *plantarum* and *S*. *aureus*, respectively. Substantive role on antibiotic resistance by the efflux‐associated genes were not confirmed, however, this study provides the experimental findings that other unknown drug resistance mechanisms may underlie the antibiotic resistance and the ethidium bromide efflux phenotype in *L*. *lactis* and *L*. *plantarum* isolates from pozol.

In vitro assays have evidenced the substantive transfer of drug‐resistant genes of lactic acid bacteria to other lactic acid bacteria and pathogenic strains like *Listeria* spp (Toomey 2009, Toomey et al. 2010). Thus, further studies focused on the phenotypic and molecular characterization of mechanism of antimicrobial resistance of *L*. *lactic* and *L*. *plantarum* may impact the safety consumption of pozol in most cases of handcraft production and in the control of the potential dissemination of antimicrobial resistance factors in foodborne pathogens.

## Conflict of Interest

None declared.
